# Barriers and Delays to Healthcare at Time of Death: Qualitative Analysis of Los Angeles County Death Records of People Experiencing Homelessness

**DOI:** 10.21203/rs.3.rs-5815264/v1

**Published:** 2025-03-26

**Authors:** Ruth A. Bishop, Christine Tarleton, Joel T. Braslow, Enrico G. Castillo

**Affiliations:** Medical University of South Carolina; University of California, Los Angeles; Columbia University Vagelos College of Physicians and Surgeons; University of California, Los Angeles

**Keywords:** Homelessness, mortality, qualitative research, barriers to care, delays in care, social determinants, health equity

## Abstract

**Background:**

Human health and homelessness are incompatible with one another. People experiencing homelessness (PEH) experience extreme health and social inequities, including a significantly higher mortality rate and lower life expectancy compared to the general adult population. While many studies have attempted to identify the most common causes of death, no study to our knowledge has sought to contextualize these deaths using death records. The objective of this study was to conduct a qualitative analysis of the Los Angeles County medical examiner records of people experiencing homelessness from 2018 in order to identify modifiable barriers and delays in accessing health care services.

**Methods:**

This study was a qualitative analysis of medical examiner records produced by the Los Angeles County Department of the Medical Examiner (DME). In 2019, the study’s senior author (EC) entered into a data use agreement with DME to provide records of deaths (n = 998) among people experiencing homelessness in 2018. The DME dataset was entered into a single file for coding using NVivo 12. Using thematic analysis as outlined by Braun and Clarke, the authors iteratively identified themes related to barriers and delays in healthcare to create a codebook.

**Results:**

A strength of this study was its identification of barriers and delays to care themes (in italics) proximal to the deaths of PEH, an outcome that community and healthcare organizations aim to reduce. PEH are often suffering from *extreme states of death due to advanced disease* and have significant *difficulty managing their health conditions,* which manifests as medication non-adherence, missed dialysis, and a lack of preventative care. These factors, as well as *disengagement and early termination of care* (declining EMS services, AMA discharges) and a *lack of a bystander response* to PEH distress may have contributed to these deaths.

**Conclusions:**

People experiencing homelessness experience many barriers and delays to care which may be linked to untimely deaths. This study highlights the importance of healthcare and community organizations serving PEH to foster social cohesion, understand reasons for PEH’s early termination and/or non-acceptance of care, and adopt equity-oriented care approaches, which aim to improve individuals’ ability to engage in outpatient services and treatment.

## INTRODUCTION

Human health and homelessness are incompatible with one another. People experiencing homelessness (PEH) experience extreme health inequities, including a significantly higher mortality rate and lower life expectancy(1–6) compared to the general adult population. Studies estimate the average lifespan of a person experiencing homelessness is reduced by 17.5 years(1), with estimates of non-elderly people experiencing homelessness having between a 1.55 to 9 times higher rate of mortality than those with housing(5–7). PEH have a higher prevalence of chronic disease and comorbid substance use and psychiatric disorders(6, 8–10), and often present with more advanced disease when compared to the general adult population(6, 11).

Barriers and delays in accessing healthcare among PEH have been conceptualized by various frameworks(11–16). One particularly extensive framework, proposed by O’Carroll et al., categorizes healthcare barriers as external or internal. External barriers include lack of health insurance(8, 17), higher rates of physical and psychiatric illness including substance use disorders(17, 18), lack of transportation(19), complexity of administrative processes(20, 21), difficulty with keeping appointments(22), lack of information on how to access services and low health literacy(22, 23), stigma and discrimination(16), and greater burden of competing priorities such as accessing food, shelter, and other essentials for survival(24–26). Internal barriers include PEH’s belief that they would not live long, denial of the severity of their health conditions, self-blame and lack of deservedness of treatment, and presumption of poor treatment or discrimination by the health system (11, 26).

Patients experiencing homelessness often lack insurance coverage in non-Medicaid expansion states due to not meeting qualifications(27) or in Medicaid-expanded states due to non-enrollment(27, 28) and therefore more often rely on acute hospital-based rather than ambulatory care (6, 8, 10, 18, 29). Institutional recidivism and disengagement with the healthcare system are common and complex(11, 30, 30), oftentimes due to multifactorial causes including both internal and external barriers, such as legacies of negative experiences with institutions, presumption of poor treatment/discrimination, feelings of fear, low self-esteem, hopelessness or embarrassment(11, 30), and untreated severe mental illness and substance use disorders(11). Additionally, demedicalization(31), which is when a health system characterizes the psychosocial needs of patients as outside their scope, further complicates access to care.

Numerous studies have attempted to better understand barriers to healthcare and identify potential interventions among people experiencing homelessness through identifying causes of death and mortality rates among PEH. Studies have shown that the most common causes of death often vary by age group or location. For example, the leading causes of death among young homeless men living in Toronto ages 18 to 24 from years 1995–1997 included accidents, overdose/poisonings, and suicides; whereas in Boston, homicide was a leading cause of death in this age group during a similar time period(32, 33). Among older men ages 45 to 64, the most common causes of death shift to chronic diseases including cancer, heart disease, overdose, and stroke(6, 32–34).

Studies characterizing homeless mortality rates often have relied on medical examiner office and state death records to determine cause of death. The Los Angeles County Department of the Medical Examiner (DME) maintains descriptions which include cause of death of people experiencing homelessness within Los Angeles county(35). From 2013–2018, the leading causes of death within Los Angeles county were coronary artery disease (22%), unintentional drug/alcohol overdose (21%), unintentional transportation-related injury (9%), homicide (6%), suicide (5%), and liver disease (5%) (35). While many studies have attempted to quantify the most common causes of death and mortality rates, no study to our knowledge has sought to contextualize these deaths using death records themselves. The situations surrounding these deaths may reveal insights into barriers and delays to care from the individual to policy level that could have contributed to premature mortality in these cases.

The objective of this study was to conduct a qualitative analysis utilizing existing frameworks, including the one proposed by O’Carroll et al., of the Los Angeles county death records of people experiencing homelessness from 2018 in order to understand the circumstances of their passing and identify potentially modifiable barriers to or delays in accessing health care services. Of note, many of the DME records often included stigmatizing language in the text, in some cases insinuating that individuals experiencing homelessness were to blame for their deaths. The authors recognize the complex interplay of structural inequities, including the barriers and delays to healthcare described within this study, and how the convergence of various inequalities contributed to PEH’s deaths.

## METHODS

This study was a qualitative analysis of medical examiner records produced by the Los Angeles County Department of the Medical Examiner (LAC DME). These records were created by DME investigators, who have a range of educational backgrounds and experiences, including former law enforcement, associate or bachelor’s degrees, and licensure as paramedics, registered nurses, and physician assistants. LAC DME investigates all deaths that are sudden, unwitnessed or unusual, due to violence, or in cases where the decedent is not under a physician’s care. DME identifies someone as being ‘homeless’ if they were found at a homeless encampment or had no known residence, whose address was that of a homeless shelter, or if death records indicated homelessness including “lives in a van” or “vagrant.”

In 2019, the study’s senior author (EC) entered into a data use agreement with DME to provide records of deaths (n = 998) among people experiencing homelessness in 2018. This dataset contains structured (non-free text) and narrative data. Each patient’s record is 150 to 250 words, and the entire dataset is 569 pages. Demographic data included age, race, gender, birth and death place, police officer contact, hospital name, laboratory samples collected, and cause of death. The dataset’s narrative fields, “Event Description” and “Synopsis,” provide descriptions of the environment where the decedent was found; medical, psychiatric, and substance use history; and evidence of illegal activity.

The DME dataset was entered into a single file for coding using NVivo 12. Using thematic analysis as outlined by Braun and Clarke(36), the senior author (EC) created codes inductively and developed initial coding categories such as “Barriers and Delays in Care” and “Social Exclusion and Inclusion.” Next, one of the authors (CT) applied the initial coding categories to a random sample of 50 cases. Study team members (CT and EC) met to refine coding categories and add subcodes. Following the initial coding of 200 cases, authors (CT and EC) created a codebook in consensus meetings to ensure a similar understanding of codes, then removed redundant codes and clarified ambiguous ones. The finalized codebook defined each code and provided examples. Study team members (CT, EC, and RB) then coded the remaining data and reviewed previously coded cases to ensure consistency.

## RESULTS

Sociodemographic data of our cohort is available in annual public reports from the LAC Department of Public Health(35). As per that report, in 2018, the same year as records in our dataset, the mortality rate among people experiencing homelessness in Los Angeles county was 1875 per 100,000 people, an increase from the 5 previous years (34). Considering race, homeless mortality rates in 2018 among White people (2013 per 100,000) were greater than that for African Americans (1074 per 100,000) and Latinos (1476 per 100,000) experiencing homelessness. However, while the rate of White deaths decreased from 2016 to 2018, the rates of death increased among African Americans and Latinos during this time frame. The leading causes of homeless deaths in Los Angeles county from 2013–2018 were coronary artery disease (22%), followed by alcohol and drug-related deaths including overdose (21%), transportation-related injury (9%), homicide (6%), suicide (5%), liver disease (5%), other unintentional injury (4%), other heart diseases including endocarditis, myocarditis, and heart failure (3%), and hypertensive heart disease (2%)(34). Men (1750 per 100,000) had higher mortality rates compared with women (1117 per 100,000). Among men, the leading cause of death was coronary artery disease whereas among women, the leading cause of death was drug and alcohol related. Adjusting for differences in demographics of the homeless population versus the general population within Los Angeles county, the all-cause mortality rate of a homeless person was estimated to be 2.3 times greater than the general population.

Building upon O’Carroll et al.’s frameworks of barriers and delays to accessing and utilizing health care among PEH, common themes centered on the high burden of complex, advanced chronic medical conditions, difficulties in accessing care and managing chronic health conditions while being homeless, lack of bystander response to PEH in crisis, and perceptions of disengagement and early termination of care among PEH.

### Extreme states of death due to advanced disease

Los Angeles county medical examiner reports describe the complexity and severity of the medical comorbidities among PEH including HIV/AIDs, diabetes complicated by renal failure or recurrent DKA, cancer, patients with amputations, spinal cord injuries and colostomy bags, and serious mental illness. Many PEH died gruesome and extreme deaths in the context of their advanced disease states. One Hispanic male with unknown past medical history was *“found deceased on the toilet in the locked restroom”* in the rear of a laundromat. A *“large amount of blood was found on the floor underneath his r[igh]t foot and his foot, sock, and shoe were blood soaked. Both lower legs were in poor condition possibly due to uncontrolled diabetes and maggots were seen to the right lower leg.”* The cause of death was listed as natural due to alcoholism and arteriosclerotic cardiovascular disease.

Another African American male with past medical history of brain tumor, pulmonary embolism, and bilateral deep venous thromboses of his legs had recently undergone craniotomy and ventriculostomy when he was found by his friend *“seated on the floor in front of a closet, unresponsive,”* just 10 days after being discharged from the hospital. Another White gentleman with past medical history of alcohol and heroin use was found *in an altered level of consciousness by EMS behind a supermarket…covered with lice and feces with areas of purulent discharge.”* He died in the ICU with severe sepsis due to cellulitis in the context of heroin and alcohol use.

Several PEH died from upper gastrointestinal bleeding in the context of cirrhosis. DME reports noted that a Hispanic male with past medical history of unspecified liver and lung issues and heavy tobacco and alcohol use who had been admitted to the hospital for hematemesis 6–8 months prior, was found unresponsive by paramedics, *“blood was noted throughout the bedroom and bathroom.”*

### Difficulty managing health conditions

Themes describing the difficulties of managing chronic diseases while experiencing homelessness highlight barriers including difficulties with access to medical services, especially when it came to filling medications, seeing a doctor, or going to dialysis. Medical examiner reports often included reports of medication non-adherence, running out of prescriptions, or filling prescriptions but not taking them at the time of death. One White male with past medical history of *“HTN, COPD, cervical fusion, nasal reconstruction surgery, smoking, and drinking” had* run out of his medications one week prior and *“had [developed] shortness of breath since.”* His shortness of breath worsened over the next several days, and he ultimately required bilevel positive airway pressure (Bipap), *“however, despite life saving measures[,] the decedent subsequently expired … [Cause of death was listed as] accident[al] methamphetamine toxicity.”*

Several reports also described non-adherence with hemodialysis in the context of PEH’s deaths. One White male with past medical history of hypertension, methamphetamine and heroin use, and ESRD on dialysis was discovered *“unresponsive in a transient encampment that he had been living in by a friend”* after he had *“stopped going to dialysis several weeks ago.”* Another Black male with past medical history of aortic valve endocarditis, hypertension, polysubstance use, systolic CHF, and ESRD who was *“non-compliant with medications and regularly misses hemodialysis”* was admitted for acute hypoxic respiratory failure, hyperkalemia, and recently diagnosed aortic valve endocarditis. He was found unresponsive while in the hospital and *“received multiple rounds of advanced cardiac life support medications”* before he was pronounced dead.

Many PEH had little contact with the healthcare system. For example, medical examiner reports mentioned how a White female with congestive heart failure, obesity, and methamphetamine use had not *“seen a doctor in over a year”* and how a White male veteran with unknown medical history was *“discovered in a storage unit”* and had *“not seen a doctor in over 20 years.”*

There were two Hispanic newborn deaths within the cohort, who were children of pregnant mothers experiencing homelessness described as having no prenatal care. One newborn’s *“mother was found under a bridge by a friend with a suspected OD on heroin”* and was found to have maternal sepsis secondary to heroin and methamphetamine overdose and placental abruption. The decedent was delivered by C-section and pronounced dead at the scene. His mother was described as receiving *“no prenatal care but would take vitamins.” Another* mother was brought to the hospital in active labor and delivered a stillborn male whom doctors believed may have been dead for some time before arrival. The newborn’s *“mother tested positive for methamphetamines and had no proof of prenatal care.”*

### Lack of bystander response

Bystanders often did not respond in a reasonable and timely manner to PEH’s signs of distress or evidence of having died. One DME report reads that *“witnesses report seeing the decedent, a […] Black male, struggling and crawling on the ground the evening before” he* was found unresponsive in a gutter with paramedics determining death on the scene. Cause of death was noted to be accidental due to cocaine intoxication. Another White male was noted to have been *“observed dec[ease]d in same position for the last three [to] four days lying on his left side naked on the front seat of his car[,] window down”* before a construction worker called paramedics who determined death on the scene due to *“atherosclerotic cardiovascular disease.”* There were also reports of PEH being found in an advanced state of decomposition, including a Hispanic female who was *“a known transient and caretaker for [a] homeowner”* who was *“discovered by homeowner yesterday but did not report it. Last seen alive 2 weeks ago. According to medical records, decedent had a recent suicide attempt via overdose.”*

### Disengagement and early termination of care

Medical examiner reports often described PEH as being medically disengaged through refusing care from paramedics or leaving hospitals against medical advice. Other forms of disengagement included medication nonadherence and inability to consistently engage with outpatient treatment or preventative care. For example, a White male was found in his vehicle in a state of decomposition *“after a welfare check was requested by a neighbor, who had not seen the decedent in two days. According to LASD’s [Los Angeles County Sheriff’s Department] interview with the neighbor, two days ago she heard knocking from inside the decedent’s vehicle, called 911, and when paramedics responded the decedent refused treatment.”*

There were also several cases of decedents who refused transport to the hospital by paramedics. For example, a Black man with *“a history of drug abuse had complained of chest and abdominal pain earlier in the day and had refused to be transported by paramedics.”* He later *“was found dead on a sidewalk near Skid Row.”*

Many people experiencing homelessness also left hospitals or emergency rooms against medical advice around their time of death. For example, *“a […] Asian male with a history of alcoholism and narcotic use for many years went to LAC [Los Angeles County] + USC [University of Southern California] medical center just prior to his death and left the facility against medical advisement.”* He was later found lying unresponsive on a sidewalk by a security guard *“with liquid coming out of his mouth.”* Paramedics were called who determined death at the scene. Another case of this was a White male with *“a history of hypertension, diabetes, congestive heart failure, high choles[e]st[e]rol, chronic obstructive pulmonary disease, chronic leg ulcers and IV, methamphetamine, heroin, and alcohol abuse [who] entered Pomona Valley Hospital Medical Center for confusion after use of heroin. He was admitted to the hospital for management of aspiration pneumonia, congestive heart failure exacerbation, and anasarca. The decedent’s medical health improved and then left the hospital without hospital consent on 07/29/2018. The next day, 07/30/2018, he was found by EMS in cardiac arrest, asystole, and PEA with an estimated down time of 25 minutes. Paramedics transported him to the hospital and CPR was initiated until a POLST [Physician Orders for Life-Sustaining Treatment] was found which was signed by the decedent to not resuscitate. Death was pronounced on 07/30/2018 at 1355 hours.”*

PEH also were noted to leave the emergency room prior to being evaluated. For example, a White male with a past medical history of anemia and diabetes mellitus *“was found unresponsive in a walkway/planter and 9-1-1 was called. Paramedic […] pronounced death on scene at 0754. The decedent was last known alive on 08/17/2018 at around 0000 when he presented to the ER of Providence St. Joseph’s medical center complaining of overall weakness. The decedent left prior to being seen by a doctor.”* The cause of death was listed as diabetic ketoacidosis.

## DISCUSSION

This qualitative study is the first of its kind, to our knowledge, utilizing death records of people experiencing homelessness from the Los Angeles County Department of the Medical Examiner in order to understand the situations in which they died and identify barriers and delays in accessing health care services. Prior qualitative studies have described numerous barriers to healthcare utilization at various levels. These include external barriers, such as physical barriers (i.e. distance), administrative barriers (i.e. appointment system, need to form and wait in lines, complexity of medical system), and attitudinal barriers (i.e. discrimination and stigma) as well as internalized barriers, including cognitive (i.e. self-blame cognitions, competing priorities cognitions, presumption of poor treatment due to past experiences) and emotional barriers (i.e. hopelessness, embarrassment), however, no clear relationship between health outcomes could necessarily be inferred from these studies. A unique strength of this study is its identification of barriers and delays to care themes proximal to the deaths of PEH, an outcome that community and healthcare organizations aim to reduce.

Analyzing 998 records, we found that people experiencing homelessness often die on the streets with advanced diseases despite having recent healthcare encounters. PEH experience difficulties in managing their chronic diseases and often die alone, later found by passersby, sometimes in a state of advanced decomposition. Lastly, reports characterize PEH’s health service utilization as chaotic; PEH are described as refusing care and then, not soon after, are found dead, or as being non-adherent to their medications or dialysis appointments.

Applying the model of health services utilization among PEH developed by O’Carroll et al., we can conceptualize our findings into external barriers (social determinants of health) and internal barriers (internalized emotions and cognitions). External barriers including serious mental illness, substance use, and lack of social support were identified within this study. While we are unable to make definitive conclusions given the nature of the dataset, one can surmise biases of some DME investigators who authored the records analyzed in this study. Prior studies have described how PEH experience considerable stigma and discrimination as external barriers (as per Canham, et al.) when seeking healthcare, and the anticipation of discrimination can be internalized by PEH and experienced as internal barriers to care seeking (as per O’Carroll, et al.); biases conveyed within the death records of PEH would be consistent with these findings(16, 18, 34). We found that some statements within death records seemed to imply that PEH were responsible for their deaths. In several instances, we found few details about DME investigators’ understanding of why PEH refused care or were non-adherent to medications or procedures. While such potential biases in these records cannot be assessed definitively, it is possible to see reflected in these records the ways that healthcare providers and systems understand PEH’s health service utilization as counter-productive, chaotic, and burdensome.

Lack of bystander intervention to PEH in crisis or to their deaths speaks to the extreme social isolation of PEH and lack of social cohesion and collective efficacy in communities where PEH live. The delays in care due to lack of social cohesion and collective efficacy are indicative of the dehumanization of PEH within communities, highlighting the critical need for evidence-based programs building social connections between community members and PEH.

Applying the concept of demedicalization(31, 37) to the data, we can identify the health systems’ pattern of demedicalizing or characterizing social needs as outside the scope of healthcare. The demedicalization of patients’ social needs and inflexibility within our current healthcare system have caused harm to PEH and even in some cases, perpetuated their homelessness. The modern day appointment-based system, decoupling of physical and mental health care, and the lack of preparedness for the health system to offer practical assistance to address social determinants of health are some examples of how our current healthcare system is better positioned to serve middle and upper class patient populations rather than marginalized ones who lack the social and monetary capital to navigate this system successfully(12).

We advocate for an equity-oriented healthcare framework as an alternative to our current system in order to better care for the complex behavioral health, physical, and social needs of PEH(38–41). An equity-oriented healthcare framework is defined as an approach that aims to reduce: 1) the effects of structural inequities, 2) the negative impacts of discrimination, racism, and stigma on people’s ability to assess services and their experiences of these services and 3) the mismatch between approaches to healthcare services (i.e. scheduled appointment model versus walk-ins) and the needs of people most affected by social and health inequities. Equity-oriented healthcare programs have been shown to increase housing stability, reduce emergency room utilization, improve health outcomes, and may even reduce healthcare costs(42–45). This model of care would address many of the barriers and delays to healthcare at time of death among PEH that were identified in this study.

Examples of health systems that have incorporated aspects of this framework include: Nationwide Children’s Hospital in Columbus, Ohio, and Bon Secours Mercy Health system in Baltimore, MD, which invested in purchasing affordable housing units and funding community projects(44); Boston Medical Center, which invested in community partnerships with affordable housing organizations(46); EQUIP Healthcare, which has multiple research and community-based projects providing resources to implement equity-oriented care in Canada(38, 47), and the Camden Coalition of Healthcare Providers in Camden, New Jersey, which provided Section 8 housing vouchers and wraparound services to reduce healthcare costs(44, 48). These various programs utilize the Housing First model, the preferred model to address chronic homelessness. Housing First provides permanent supportive housing without prerequisites to engage in substance use or behavioral health treatment prior to getting housed. These examples of investment in affordable housing and hospital-housing partnerships are just a few models of how health systems can incorporate the equity-oriented healthcare framework into their organization to better address barriers and delays in care among PEH.

This study confirms that homelessness and poor health are deeply connected, with homelessness exacerbating poor health conditions, and health issues being a risk factor for homelessness. PEH experience many barriers and delays to care which may be linked to their untimely deaths. Without housing, PEH have significantly more difficulties in managing their chronic health conditions and staying engaged (and therefore adherent) with their care. An equity-oriented healthcare approach provides a path forward for both institutions and policy makers to improve health outcomes among this vulnerable patient population.

## LIMITATIONS

This study has several limitations. This was a qualitative study utilizing records written by Los Angeles county DME investigators, so these records may contain biases and inaccuracies regarding the circumstances at time of death. These records are also limited given that the person experiencing homelessness was unable to discuss their perceptions of barriers and delays to accessing healthcare. Therefore, this study was limited in its ability to capture certain kinds of barriers and delays of care, specifically internal cognitions and behaviors (such as a PEH’s perception of stigma), given that these records were third party accounts. Additionally, this study may not be generalizable to other geographic locations or marginalized patient populations.

## CONCLUSIONS

Implications for Healthcare and Community Organizations Serving People Experiencing Homelessness

A strength of this study was its identification of barriers and delays to care themes (in italics) proximal to the deaths of PEH, an outcome that community and healthcare organizations aim to reduce. PEH are often suffering from *extreme states of death due to advanced disease* and have significant *difficulty managing their health conditions,* which manifests as medication non-adherence, missed dialysis, and a lack of preventative care. These factors, as well as *disengagement and early termination of care* (declining EMS services, AMA discharges) and a *lack of a bystander response* to PEH distress may have contributed to these deaths. This study highlights how healthcare systems’ demedicalization of the social determinants of health may result in barriers and delays in care among PEH. The implications of this work for healthcare and community organizations serving PEH is to highlight the need to foster social cohesion, understand reasons for PEH’s care refusals, and adopt equity-oriented care and Housing First approaches, which aim to improve individuals’ ability to engage in treatment.

## Data Availability

The datasets generated and/or analyzed during the current study are not publicly available due to the terms of our study team’s data use agreement with the County of Los Angeles Medical Examiner but are available to third party researchers directly from the Medical Examiner’s Office.

## Abbreviations

PEH: People Experiencing Homelessness
